# From Liver to Lung: A Pathological Journey of Bilothorax

**DOI:** 10.7759/cureus.100382

**Published:** 2025-12-29

**Authors:** Momina Abid, Mohammad T Hussain, Sofia Barlas, Hassan Z Baig

**Affiliations:** 1 Medicine, University Medical and Dental College, Faisalabad, PAK; 2 Internal Medicine, Mayo Clinic, Jacksonville, USA; 3 Osteopathic Medicine, New York Institute of Technology College of Osteopathic Medicine, New York, USA; 4 Pulmonary Medicine, Mayo Clinic, Jacksonville, USA

**Keywords:** abdominal sepsis, advanced liver disease, bile pleural effusion, biliary peritonitis, bilothorax, chest tube drainage, cirrhosis, hepatocellular carcinoma, thoracic empyema

## Abstract

Bilothorax is a rare and pathological condition characterized by the accumulation of bile in the pleural cavity. This phenomenon most commonly occurs on the right side due to anatomical proximity to the liver and biliary system. Bilateral or left-sided bilothorax remains exceedingly rare. Diagnosis relies on pleural fluid analysis, particularly a pleural-to-serum bilirubin ratio >1, and imaging.

We report the case of a male patient in his mid-60s with hepatitis C-related cirrhosis (Child-Pugh B7), hepatocellular carcinoma with pulmonary metastases, and multiple comorbidities, including diabetes mellitus and portal hypertension. The patient was undergoing systemic therapy with atezolizumab and bevacizumab, presented with chronic infected wounds and systemic symptoms and later developed gallbladder perforation with biliary peritonitis. Despite multidisciplinary management including percutaneous drainage, intravenous antibiotics, and intensive care support, his condition deteriorated. Subsequently, he developed a right-sided bilothorax, confirmed via thoracentesis, with a pleural-to-serum bilirubin ratio of 3.4 (normal < 0.6). Cultures isolated Enterobacter, and the patient was treated with targeted antibiotics and chest tube drainage. However, he experienced progressive hepatic decompensation, recurrent sepsis, and respiratory failure, ultimately leading to a transition to hospice care. This case is noteworthy due to the extreme rarity of bilothorax, the diagnostic complexity in distinguishing it from other pleural effusions, and the management challenges it poses in patients with advanced cirrhosis and malignancy.

## Introduction

Bilothorax, or thoraco-bilia, is a rare pathological condition defined as the accumulation of bile within the pleural cavity, most commonly resulting from an abnormal communication between the biliary system and the pleural space. This communication may arise secondary to diaphragmatic defects, traumatic or iatrogenic injury, or the formation of a bilio-pleural or pleuro-biliary fistula following hepatobiliary interventions such as percutaneous transhepatic biliary drainage or surgery. Under normal conditions, there is no anatomical continuity between the abdominal and pleural compartments; thus, the presence of bilirubin in the pleural fluid is always pathological [1]. The first reported case of bilothorax was in a young man who developed right-sided bilothorax following blunt trauma in 1971 [2]. Since then, fewer than 100 cases have been documented in the literature, highlighting its rarity [1]. Most of these cases are presented on the right side because of the proximity and anatomic location to the liver and biliary system, while bilateral cases are extremely rare [2]. They are often associated with malignant or benign biliary obstruction, pleuro-biliary fistulas after hepatobiliary procedures, i.e., surgery or percutaneous drainage, and hepatic or subphrenic abscesses [2,3]. Nevertheless, this represents a serious complication, as it can progress to thoracic empyema. Therefore, maintaining a high index of clinical suspicion is essential.

## Case presentation

A male patient in his mid-60s with a history of hepatitis C-related cirrhosis (Child-Pugh B7) complicated by hepatocellular carcinoma with metastasis to the lungs was under active oncologic management with atezolizumab and bevacizumab. He is a known case of diabetes mellitus and portal hypertension. The patient had previously undergone variceal banding, esophagogastroduodenoscopy, and hepatic dome Y-90 radioembolization and was considered ineligible for transplantation due to metastatic disease. Imaging revealed cirrhosis with portal hypertension, suspicious new hepatic lesions, 7 mm anterior mediastinal and 4 mm pulmonary nodules, and a small anterior mediastinal lymph node.

He presented with painful chronic wounds over the left ankle and gluteal region, associated with fatigue, subjective fevers, and poor diabetic control. Examination and imaging raised concern for an infected gluteal cyst versus a metastatic lesion; labs showed total bilirubin 12.3 mg/dl (reference range: 0.3-1.2 mg/dL), total protein 8.5 g/dl (reference range: 6.0-8.3 g/dL), and albumin 3.9 g/dl (reference range: 3.5-5.0 g/dL). He was hospitalized and started on intravenous antibiotics. During hospitalization, he developed acute abdominal pain and CT with IV contrast was obtained that revealed gallbladder perforation with peritonitis, diffuse colitis/ileitis, and findings concerning for left gluteal subcutaneous abscess, ultrasound was also obtained that confirmed diagnosis of perforated acute cholecystitis surgical team was consulted for perforated acute cholecystitis and ICU team was consulted for evaluation due to concern for high risk for clinical decompensation, appeared toxic and in severe peritoneal pain patient was transferred to the ICU. Given the concern for perforated cholecystitis with peritonitis, worsening lactic acidosis, and impending septic shock, the patient was transferred to the ICU, where broad-spectrum antibiotics were initiated. The Colorectal Surgery Team and Gastroenterology team were consulted, who recommended abdominal drainage with a catheter inserted for his ascites. 

Interventional radiology placed a right-sided abdominal drain, which produced bile-stained fluid, confirming gallbladder perforation with biliary peritonitis. A second pigtail drain was subsequently placed into a perihepatic collection, and the pleural fluid cultures grew E. coli. Additional drains were inserted in the right posterior flank and right lower quadrant. At the same time, the patient developed an ischio-gluteal abscess, which was drained, and wound cultures were sent. Despite drainage and antimicrobial therapy, serial CT imaging demonstrated persistent perihepatic collections, diffuse thickening of the small and large intestines, and severe colitis. The etiology of bowel thickening remained unclear, with considerations including portal hypertension, ischemia, infection, or inflammatory bowel disease. Because of advanced cirrhosis and metastatic disease, the surgical and GI teams did not recommend endoscopy or operative intervention, citing high perioperative risk (30-day mortality 8%, 90-day decompensation 38%).

**Figure 1:**
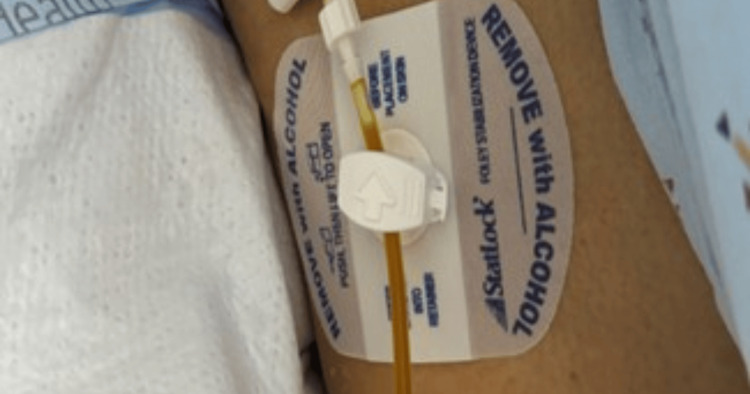
Chest tube exit site with dark green bilious fluid.

**Figure 2:**
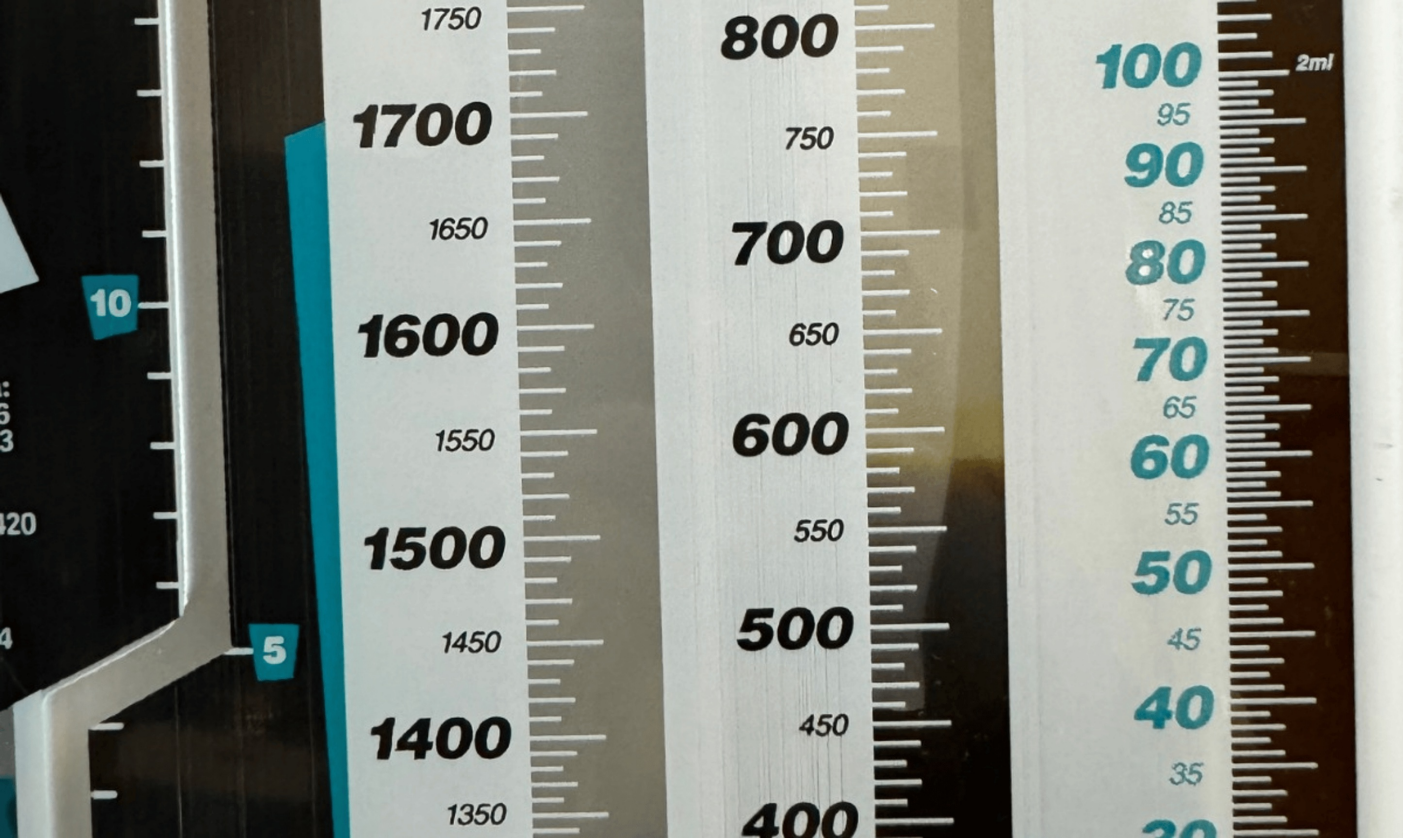
600–900 mL of dark green bilious fluid.

The patient’s hospital course was complicated by recurrent biliary ascites and, later, the development of a right-sided bilothorax. He subsequently underwent thoracentesis, draining 600-900 mL of dark green, bilious fluid on each occasion. The pleural fluid analysis revealed a WBC count of 12,000/mm³ (reference range: <1,000 cells/mm³) with a markedly elevated bilirubin level of 52.4 mg/dL, and a pleural bilirubin to serum bilirubin ratio being 3.4 (reference range: <1), fulfilling diagnostic criteria for bilothorax (Figures 1, 2).

**Figure 3:**
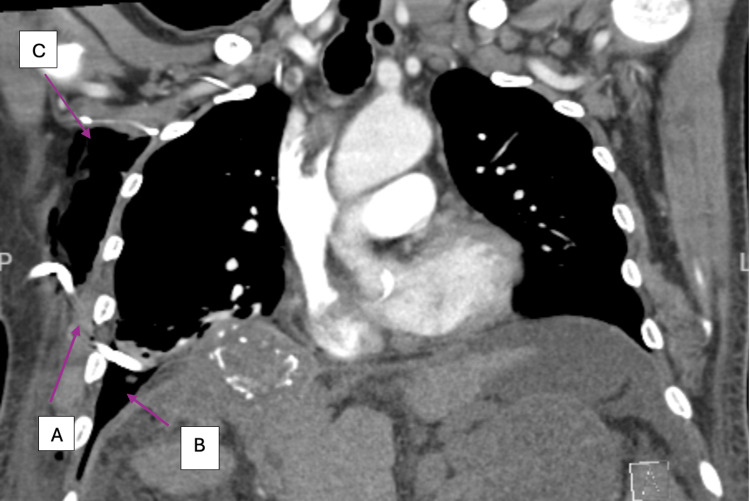
CT scan of the chest (lung view): (A) Chest tube in place and (B) hydropneumothorax with small left pleural effusion and near complete atelectasis of the right lower lobe. (C) Moderate amount of right chest wall subcutaneous emphysema.

**Figure 4:**
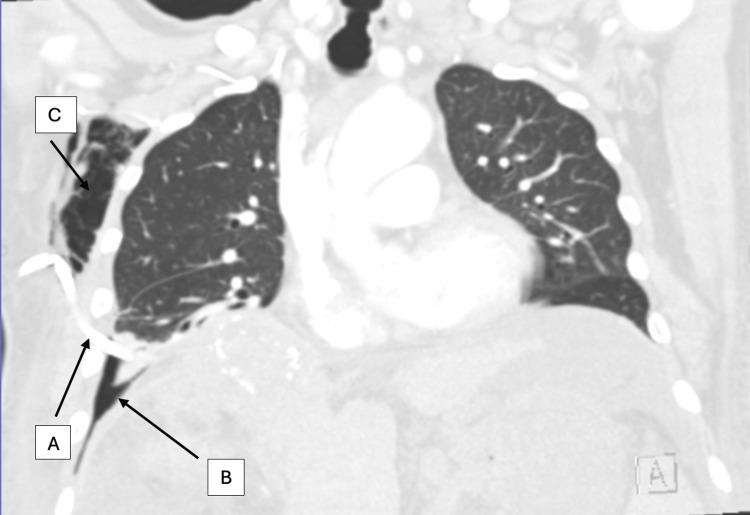
CT scan of the chest (lung view): (A) Chest tube in place and (B) hydropneumothorax with small left pleural effusion and near complete atelectasis of the right lower lobe. (C) Moderate amount of right chest wall subcutaneous emphysema.

Cultures eventually grew Enterobacter, and he was treated with intravenous levofloxacin; vancomycin was discontinued. A 14 French chest tube was inserted for continuous drainage. Post-procedure chest X-rays showed improving effusion, but persistent right basilar pneumothorax with subcutaneous emphysema, and a CT scan of the chest showed a small right-sided hydropneumothorax with a right basilar chest tube in place and a small left pleural effusion with partial atelectasis of the left lower lobe and near complete atelectasis of the right lower lobe (Figures 3, 4).

Throughout admission, laboratory studies demonstrated worsening synthetic liver failure with INR 2, total bilirubin 15.3 mg/dL, direct bilirubin 12 mg/dL (reference range: 0.0-0.3 mg/dL), albumin 2.8 g/dL, and elevated lactate at 3.3 mmol/L (reference range: 0.5-2.2 mmol/L). Imaging also revealed progressive ascites, right middle-lobe atelectasis, and bibasilar consolidations. Despite NG decompression, broad-spectrum antibiotics (Unasyn followed by Augmentin), and multiple drainage procedures, the patient’s condition progressively declined. He remained on broad-spectrum antibiotics and bowel decompression.

Given worsening empyema, recurrent sepsis, and respiratory distress in the setting of cirrhosis, hepatocellular carcinoma, acute kidney injury, and profound deconditioning, goals-of-care discussions were held. Given his advanced liver disease and high operative risk, surgical and interventional teams recommended nonoperative management. The patient elected for DNR/DNI status, and with the support of his daughter and son, transitioned to inpatient hospice.

## Discussion

Bilothorax is considered one of the causes of black pleural effusion [4]. Leakage of bile into the pleural cavity may develop when inflammatory processes erode the diaphragm, such as in cases of hepatic abscess or tumor infiltration. In addition, strictures of the biliary ducts, whether due to malignancy, fibrotic changes, or granulomatous disease, can give rise to bilomas, which, if sufficiently large, may breach the diaphragm [5].

The exact mechanism by which bile enters the pleural space remains a matter of debate; however, several plausible routes have been proposed. One suggested pathway involves the passive movement of bile through the diaphragm, similar to the mechanism seen in hepatic hydrothorax. In this scenario, increased negative intrathoracic pressure facilitates the translocation of bile through microscopic diaphragmatic pores. Another proposed mechanism involves lymphatic drainage, where bile-containing fluid collected in the subphrenic space is transported through peritoneal lymphatics into pleural lymphatics via pleuroperitoneal lymphatic connections [6].

Bile acids act as strong chemical irritants, and when biliary contents enter the thoracic cavity, they trigger marked inflammation. Patients with bilothorax typically present with symptoms such as pleuritic pain on the affected side, shortness of breath, low oxygen saturation, and, in severe cases, respiratory compromise. Because bile provides a favorable environment for microbial proliferation, these patients are particularly vulnerable to secondary bacterial empyema and translocation of organisms from the gastrointestinal tract [4]. The perforation results in mediastinitis, pleural collections, and sepsis [2,4].

Literature shows dual diagnosis of combined empyema and bilothorax. Published case reports and series demonstrate that pleural fluid findings are heterogeneous. Some patients present with frank empyema, others with bilious exudates, and a few with complex or loculated effusions [6]. In our patient, pleural fluid analysis revealed a bilious exudate with a pleural-to-serum bilirubin ratio of 3.4, consistent with previously reported cases. This highlights the value of this ratio as a diagnostic marker, particularly when the gross appearance of the fluid may overlap with other effusions.

The organisms responsible for complicated and empyematous bilious effusions are typically derived from the gastrointestinal tract. Common pathogens include Enterobacter species, Enterococcus faecalis, and Staphylococcus aureus, with Escherichia coli being the most frequently isolated organism, followed by Klebsiella species [6,7].

The establishment of diagnosis in cases of bilious pleural effusion can be achieved with high sensitivity through two straightforward assessments: identifying glycocholic acid within the pleural fluid and demonstrating a pleural fluid-to-serum total bilirubin ratio exceeding one. A study demonstrated that using the pleural-to-serum T-Bil ratio >1 alone yields a sensitivity of 76.9% [4] for detecting bilious pleural effusion. In our patient, the ratio was 3.4, confirming the diagnosis of bilothorax. This sensitivity increases to 100% [4] when combined with the detection of glycoholic acid, the primary component of bile acids, in the pleural fluid. Pleural fluid-to-serum bilirubin ratios in a case series conducted with 52 reported cases from 1960 to 2016 were greater than 1.0, with a range of 1.4 to 11.6 [6]. In addition to these biochemical tests, imaging modalities such as chest radiography, ultrasound, and computed tomography (CT) can aid in establishing an accurate clinical diagnosis [4,6].

Early administration of broad-spectrum antibiotics and prompt drainage of the pleural space are essential to prevent infection and complications in patients with bilious pleural effusion. The mainstay of treatment involves chest tube drainage of the bilothorax, which is effective in most cases. However, if the effusion does not resolve with chest tube placement alone, surgical intervention may be required. Approximately 45% [6] of patients have been reported to need surgical procedures, with biliary decompression being the most common. Advances in minimally invasive techniques, such as percutaneous biliary drainage and endoscopic retrograde cholangiopancreatography (ERCP), play a crucial role in relieving biliary obstruction and complement pleural drainage in the overall management strategy [5-7].

This case underscores the importance of early recognition of bilothorax, particularly in patients with complex hepatobiliary disease, as prompt diagnosis allows for timely interventions such as chest tube drainage and targeted antibiotics, which can prevent sepsis and reduce morbidity. In patients with advanced liver disease and metastatic malignancy, procedural risks are significant, and management must be individualized. Our patient’s care highlights the need to balance aggressive supportive measures with the patient’s overall prognosis, comorbidities, and goals of care. Palliative discussions, including consideration of DNR/DNI status and transition to hospice, are essential components of management in high-risk patients, ensuring that clinical decisions align with patient and family preferences while optimizing quality of life.

## Conclusions

Bilothorax is a rare but serious form of exudative pleural effusion, typically resulting from biliary obstruction or injury. Diagnosis requires a high index of suspicion, particularly in patients with recent hepatobiliary surgery, trauma, infection, or interventions. A pleural fluid-to-serum bilirubin ratio greater than one is considered the most specific diagnostic marker. Management can be challenging, and while treatment strategies are based largely on case reports and small series, early recognition, appropriate pleural drainage, and management of the underlying biliary pathology are critical to improving outcomes in this uncommon but potentially life-threatening condition. In complex hepatobiliary cases, clinical decision-making may involve minimally invasive interventions such as ERCP or percutaneous biliary drainage, and in selected patients, surgical decompression. Furthermore, palliative considerations should be incorporated in patients with advanced disease or limited physiological reserve to balance procedural benefits and overall quality of life. Overall, awareness of this uncommon but potentially life-threatening condition is essential for optimizing patient outcomes and guiding individualized management strategies.
